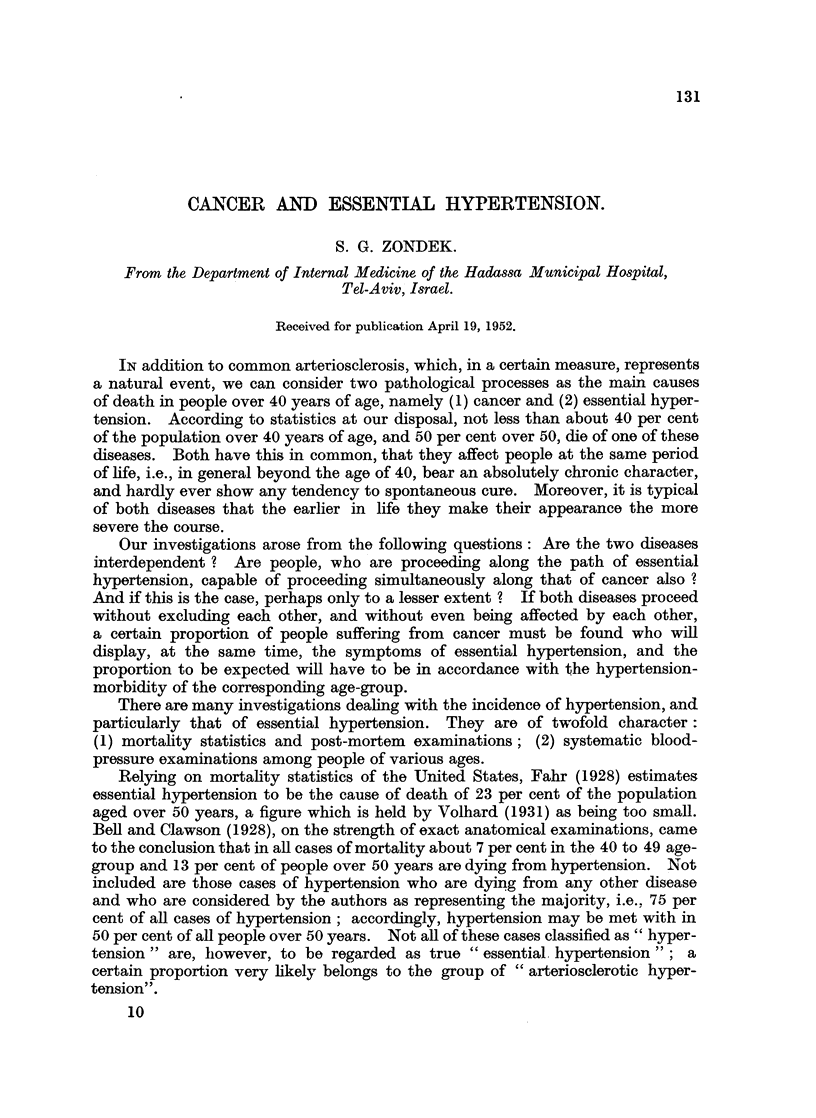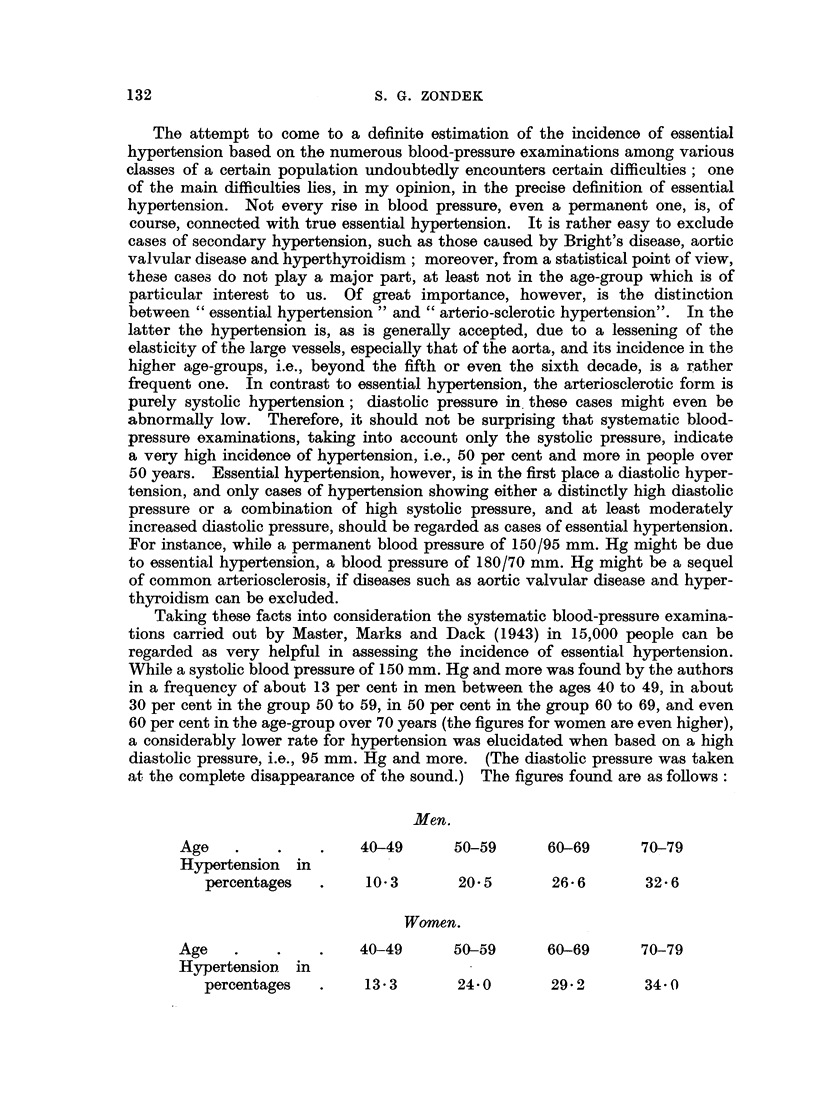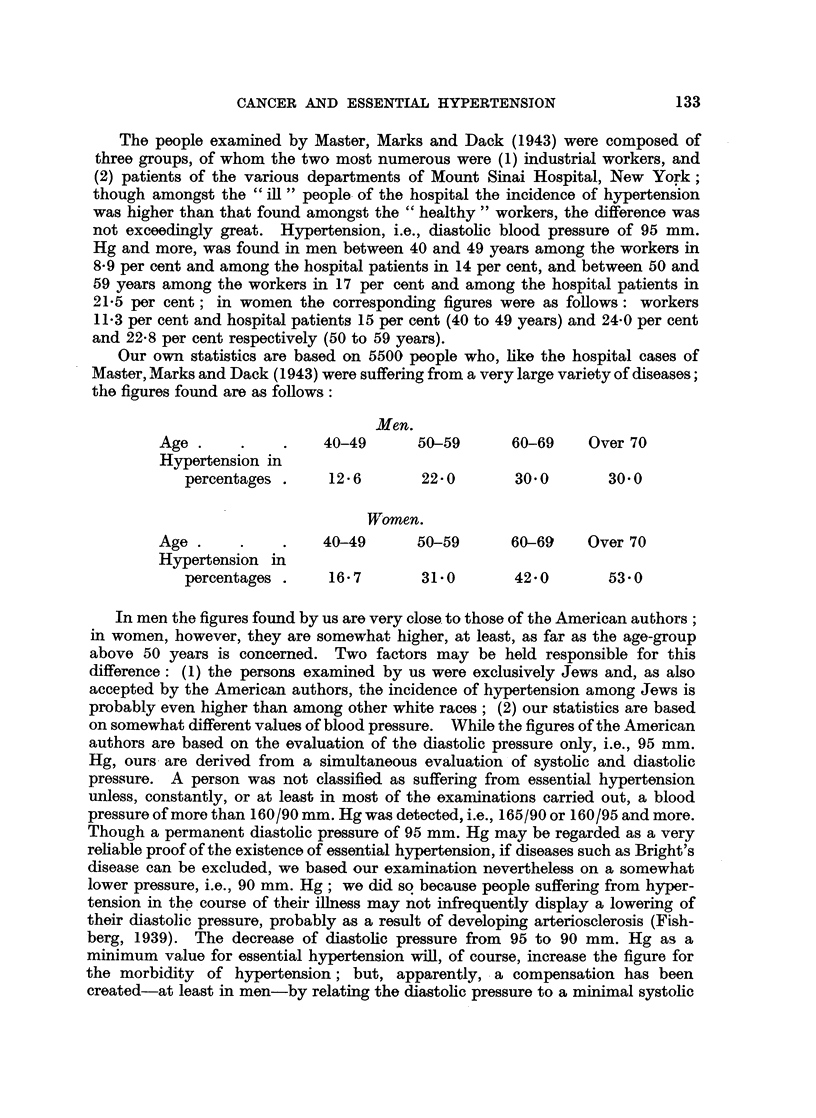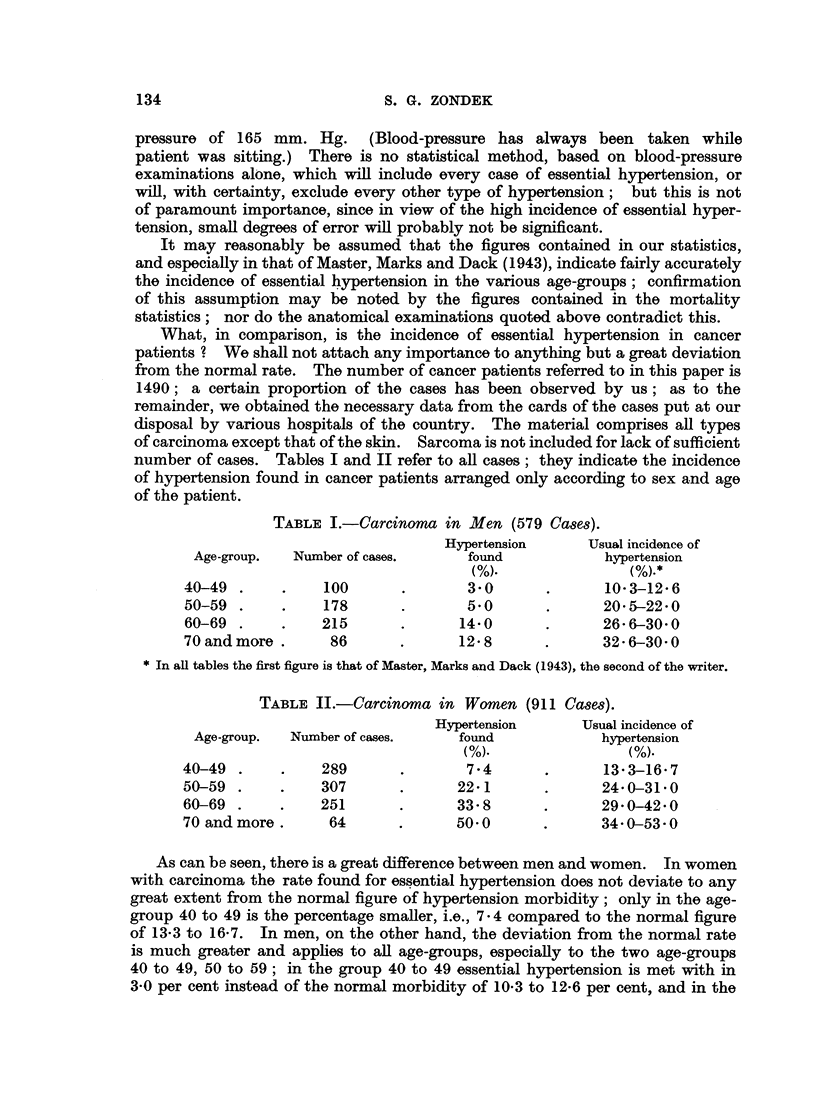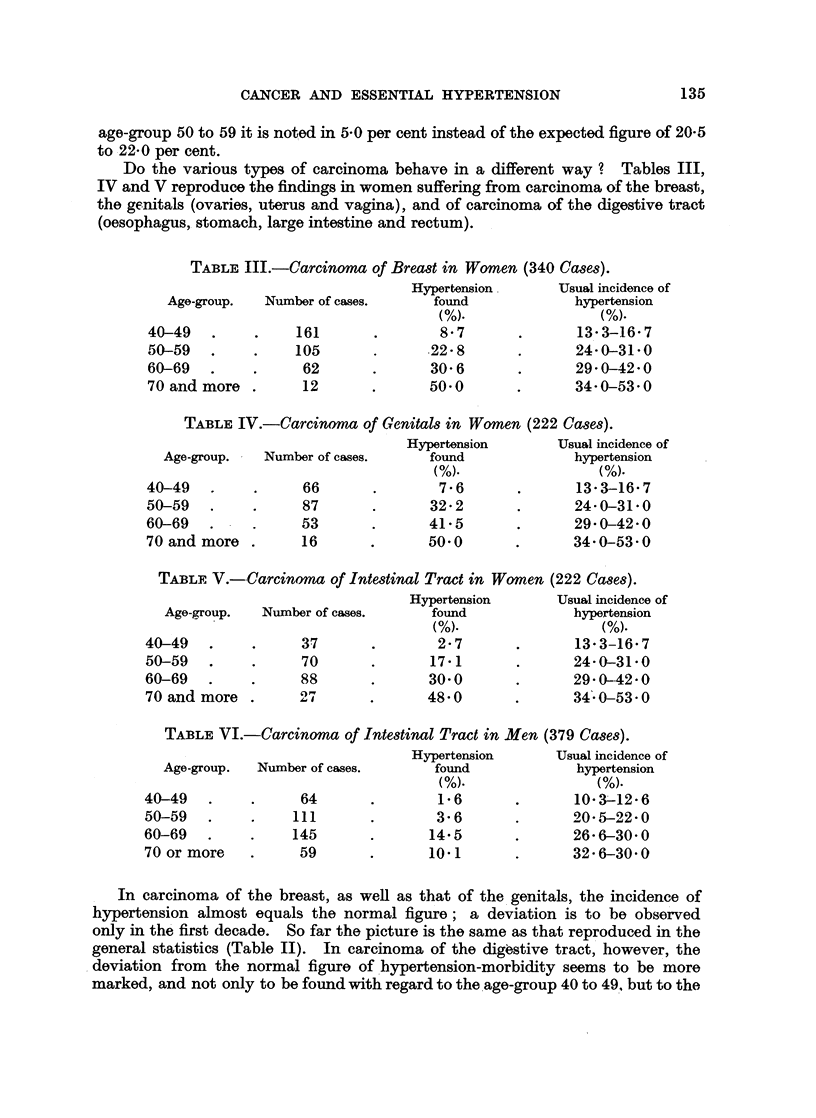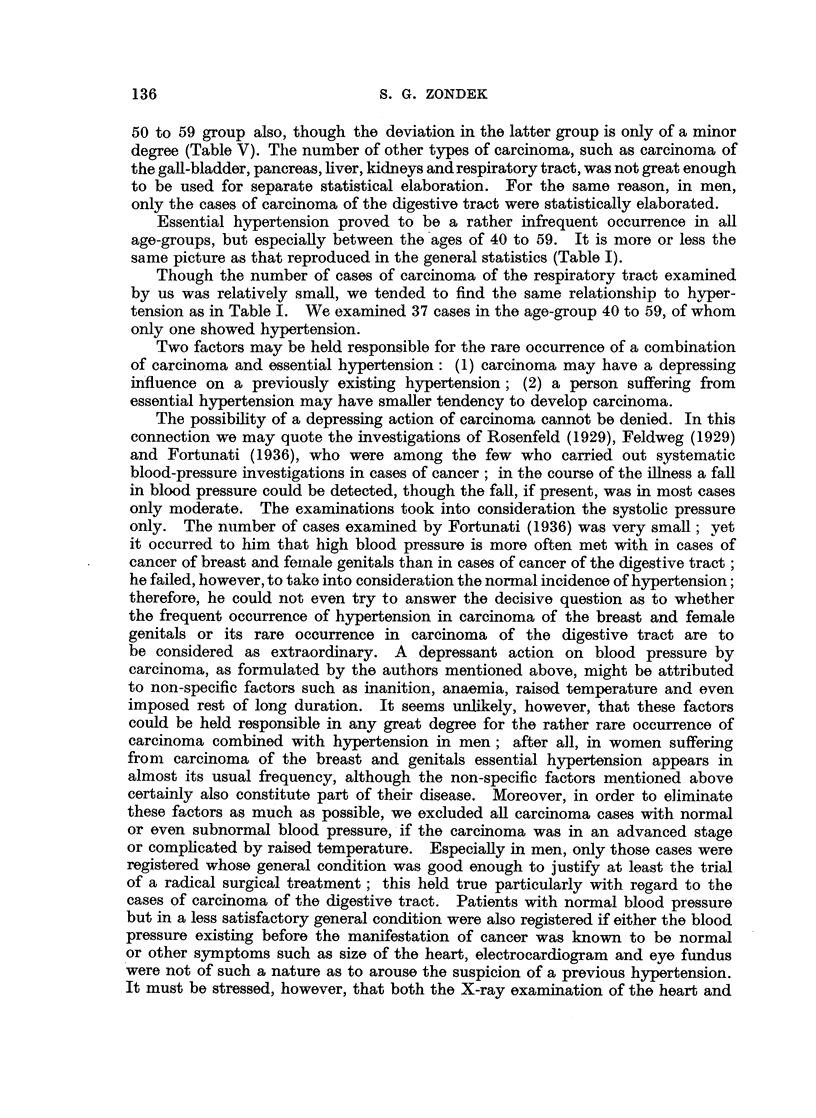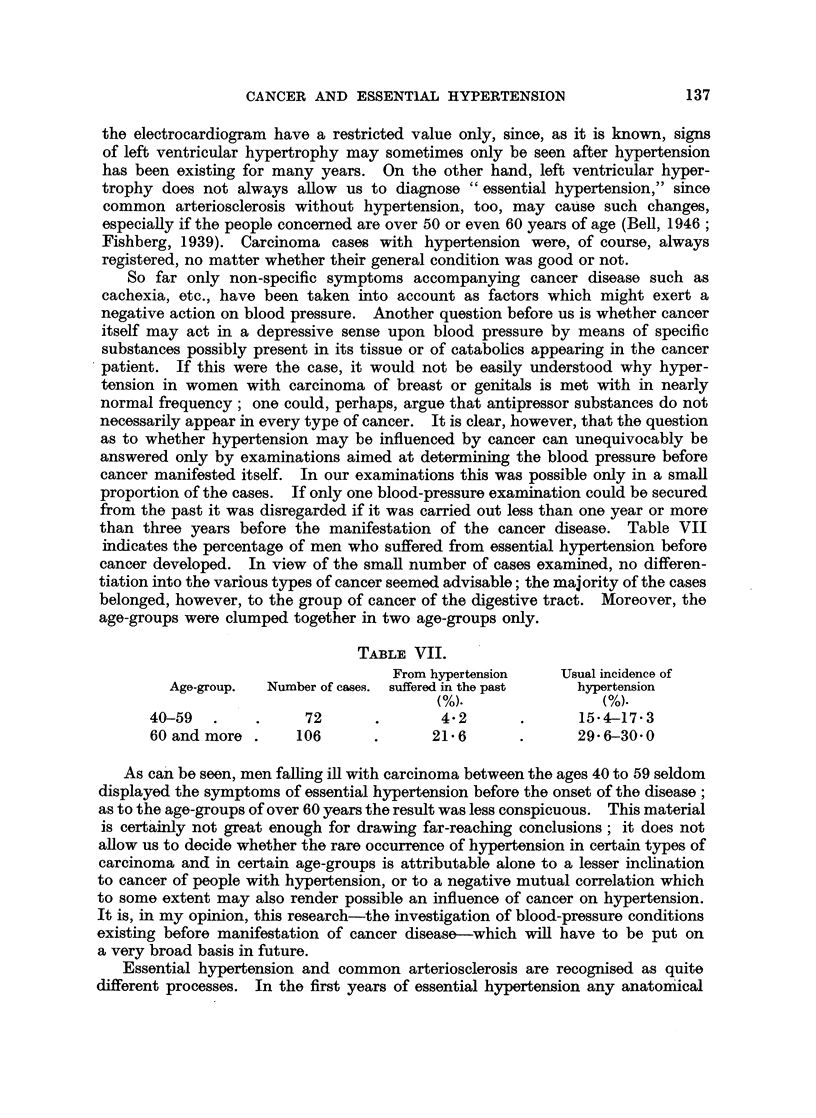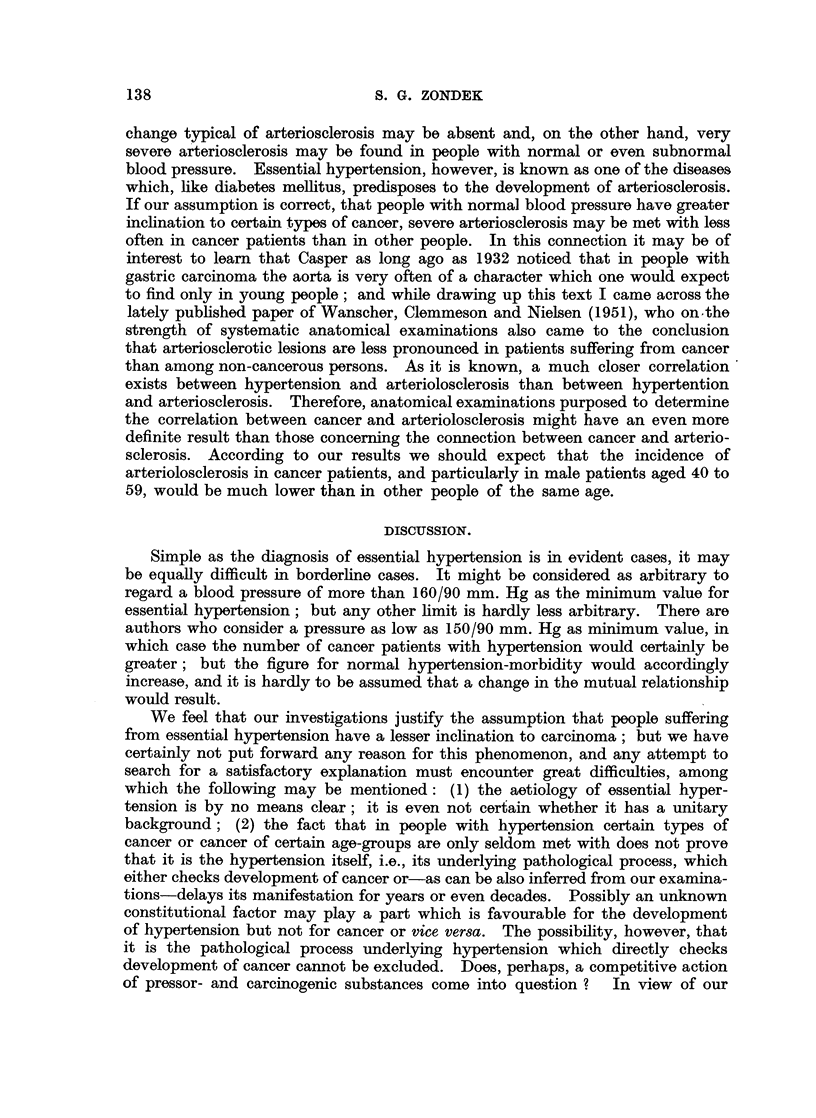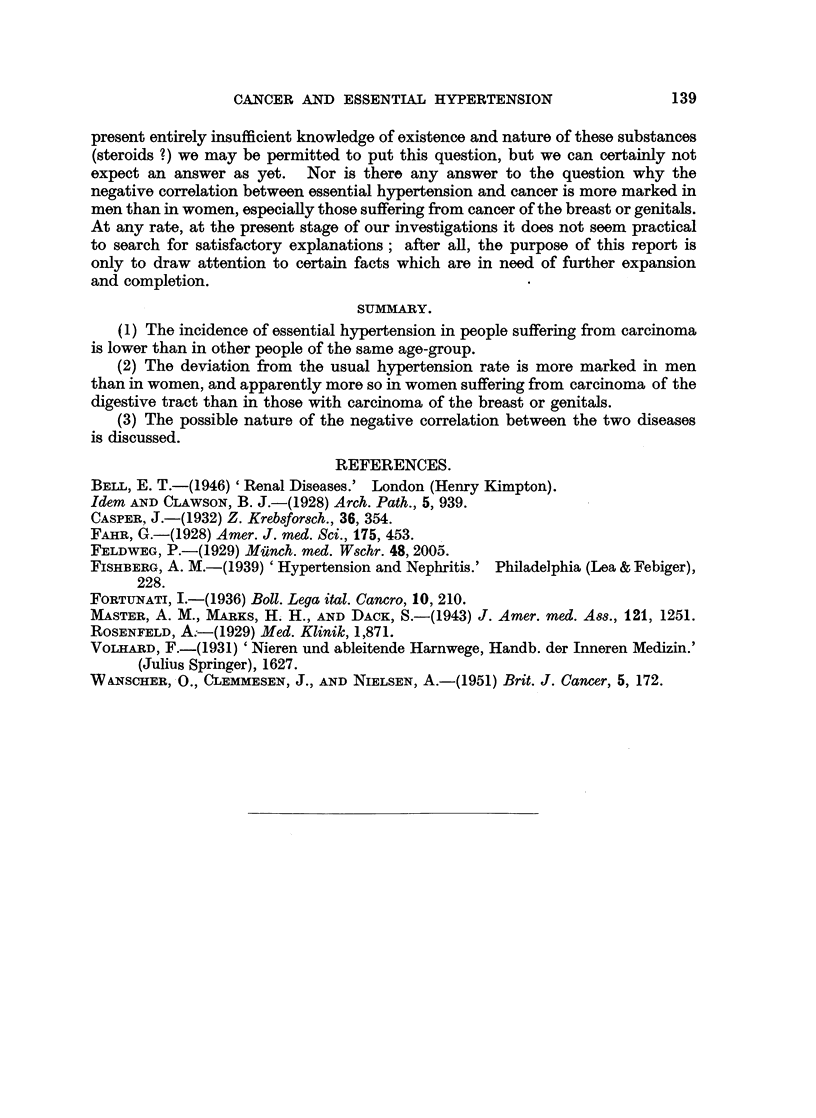# Cancer and Essential Hypertension

**DOI:** 10.1038/bjc.1952.15

**Published:** 1952-06

**Authors:** S. G. Zondek


### CANCER AND ESSENTIAL HYPERTENSION.

S. G. ZONDEK.

*From the Department of Internal Medicine of the Hadassa Municipal Hospital, Tel-Aviv, Israel.*

Received for publication April 19, 1952.

IN addition to common arteriosclerosis, which, in a certain measure, represents a natural event, we can consider two pathological processes as the main causes of death in people over 40 years of age, namely (1) cancer and (2) essential hypertension. According to statistics at our disposal, not less than about 40 per cent of the population over 40 years of age, and 50 per cent over 50, die of one of these diseases. Both have this in common, that they affect people at the same period of life, i.e., in general beyond the age of 40, bear an absolutely chronic character, and hardly ever show any tendency to spontaneous cure. Moreover, it is typical of both diseases that the earlier in life they make their appearance the more severe the course.

Our investigations arose from the following questions : Are the two diseases interdependent ? Are people, who are proceeding along the path of essential hypertension, capable of proceeding simultaneously along that of cancer also ? And if this is the case, perhaps only to a lesser extent ? If both diseases proceed without excluding each other, and without even being affected by each other, a certain proportion of people suffering from cancer must be found who will display, at the same time, the symptoms of essential hypertension, and the proportion to be expected will have to be in accordance with the hypertension-morbidity of the corresponding age-group.

There are many investigations dealing with the incidence of hypertension, and particularly that of essential hypertension. They are of twofold character : (1) mortality statistics and post-mortem examinations ; (2) systematic blood-pressure examinations among people of various ages.

Relying on mortality statistics of the United States, Fahr (1928) estimates essential hypertension to be the cause of death of 23 per cent of the population aged over 50 years, a figure which is held by Volhard (1931) as being too small. Bell and Clawson (1928), on the strength of exact anatomical examinations, came to the conclusion that in all cases of mortality about 7 per cent in the 40 to 49 age-group and 13 per cent of people over 50 years are dying from hypertension. Not included are those cases of hypertension who are dying from any other disease and who are considered by the authors as representing the majority, i.e., 75 per cent of all cases of hypertension ; accordingly, hypertension may be met with in 50 per cent of all people over 50 years. Not all of these cases classified as " hypertension " are, however, to be regarded as true " essential hypertension " ; a certain proportion very likely belongs to the group of " arteriosclerotic hypertension".

The attempt to come to a definite estimation of the incidence of essential hypertension based on the numerous blood-pressure examinations among various classes of a certain population undoubtedly encounters certain difficulties ; one of the main difficulties lies, in my opinion, in the precise definition of essential hypertension. Not every rise in blood pressure, even a permanent one, is, of course, connected with true essential hypertension. It is rather easy to exclude cases of secondary hypertension, such as those caused by Bright's disease, aortic valvular disease and hyperthyroidism ; moreover, from a statistical point of view, these cases do not play a major part, at least not in the age-group which is of particular interest to us. Of great importance, however, is the distinction between " essential hypertension " and " arterio-sclerotic hypertension". In the latter the hypertension is, as is generally accepted, due to a lessening of the elasticity of the large vessels, especially that of the aorta, and its incidence in the higher age-groups, i.e., beyond the fifth or even the sixth decade, is a rather frequent one. In contrast to essential hypertension, the arteriosclerotic form is purely systolic hypertension ; diastolic pressure in these cases might even be abnormally low. Therefore, it should not be surprising that systematic blood-pressure examinations, taking into account only the systolic pressure, indicate a very high incidence of hypertension, i.e., 50 per cent and more in people over 50 years. Essential hypertension, however, is in the first place a diastolic hypertension, and only cases of hypertension showing either a distinctly high diastolic pressure or a combination of high systolic pressure, and at least moderately increased diastolic pressure, should be regarded as cases of essential hypertension. For instance, while a permanent blood pressure of 150/95 mm. Hg might be due to essential hypertension, a blood pressure of 180/70 mm. Hg might be a sequel of common arteriosclerosis, if diseases such as aortic valvular disease and hyperthyroidism can be excluded.

Taking these facts into consideration the systematic blood-pressure examinations carried out by Master, Marks and Dack (1943) in 15,000 people can be regarded as very helpful in assessing the incidence of essential hypertension. While a systolic blood pressure of 150 mm. Hg and more was found by the authors in a frequency of about 13 per cent in men between the ages 40 to 49, in about 30 per cent in the group 50 to 59, in 50 per cent in the group 60 to 69, and even 60 per cent in the age-group over 70 years (the figures for women are even higher), a considerably lower rate for hypertension was elucidated when based on a high diastolic pressure, i.e., 95 mm. Hg and more. (The diastolic pressure was taken at the complete disappearance of the sound.) The figures found are as follows :

*Men.*

| Age . . . | 40–49 | 50–59 | 60–69 | 70–79 |
|---|---|---|---|---|
| Hypertension in percentages . | 10·3 | 20·5 | 26·6 | 32·6 |

*Women.*

| Age . . . | 40–49 | 50–59 | 60–69 | 70–79 |
|---|---|---|---|---|
| Hypertension in percentages . | 13·3 | 24·0 | 29·2 | 34·0 |

The people examined by Master, Marks and Dack (1943) were composed of three groups, of whom the two most numerous were (1) industrial workers, and (2) patients of the various departments of Mount Sinai Hospital, New York; though amongst the "ill" people of the hospital the incidence of hypertension was higher than that found amongst the "healthy" workers, the difference was not exceedingly great. Hypertension, i.e., diastolic blood pressure of 95 mm. Hg and more, was found in men between 40 and 49 years among the workers in 8·9 per cent and among the hospital patients in 14 per cent, and between 50 and 59 years among the workers in 17 per cent and among the hospital patients in 21·5 per cent; in women the corresponding figures were as follows: workers 11·3 per cent and hospital patients 15 per cent (40 to 49 years) and 24·0 per cent and 22·8 per cent respectively (50 to 59 years).

Our own statistics are based on 5500 people who, like the hospital cases of Master, Marks and Dack (1943) were suffering from a very large variety of diseases; the figures found are as follows:

*Men.*

| | | | | |
|---|---|---|---|---|
| Age . . . | 40–49 | 50–59 | 60–69 | Over 70 |
| Hypertension in percentages . | 12·6 | 22·0 | 30·0 | 30·0 |

*Women.*

| | | | | |
|---|---|---|---|---|
| Age . . . | 40–49 | 50–59 | 60–69 | Over 70 |
| Hypertension in percentages . | 16·7 | 31·0 | 42·0 | 53·0 |

In men the figures found by us are very close to those of the American authors; in women, however, they are somewhat higher, at least, as far as the age-group above 50 years is concerned. Two factors may be held responsible for this difference: (1) the persons examined by us were exclusively Jews and, as also accepted by the American authors, the incidence of hypertension among Jews is probably even higher than among other white races; (2) our statistics are based on somewhat different values of blood pressure. While the figures of the American authors are based on the evaluation of the diastolic pressure only, i.e., 95 mm. Hg, ours are derived from a simultaneous evaluation of systolic and diastolic pressure. A person was not classified as suffering from essential hypertension unless, constantly, or at least in most of the examinations carried out, a blood pressure of more than 160/90 mm. Hg was detected, i.e., 165/90 or 160/95 and more. Though a permanent diastolic pressure of 95 mm. Hg may be regarded as a very reliable proof of the existence of essential hypertension, if diseases such as Bright's disease can be excluded, we based our examination nevertheless on a somewhat lower pressure, i.e., 90 mm. Hg; we did so because people suffering from hypertension in the course of their illness may not infrequently display a lowering of their diastolic pressure, probably as a result of developing arteriosclerosis (Fishberg, 1939). The decrease of diastolic pressure from 95 to 90 mm. Hg as a minimum value for essential hypertension will, of course, increase the figure for the morbidity of hypertension; but, apparently, a compensation has been created—at least in men—by relating the diastolic pressure to a minimal systolic pressure of 165 mm. Hg. (Blood-pressure has always been taken while patient was sitting.) There is no statistical method, based on blood-pressure examinations alone, which will include every case of essential hypertension, or will, with certainty, exclude every other type of hypertension ; but this is not of paramount importance, since in view of the high incidence of essential hypertension, small degrees of error will probably not be significant.

It may reasonably be assumed that the figures contained in our statistics, and especially in that of Master, Marks and Dack (1943), indicate fairly accurately the incidence of essential hypertension in the various age-groups ; confirmation of this assumption may be noted by the figures contained in the mortality statistics ; nor do the anatomical examinations quoted above contradict this.

What, in comparison, is the incidence of essential hypertension in cancer patients ? We shall not attach any importance to anything but a great deviation from the normal rate. The number of cancer patients referred to in this paper is 1490 ; a certain proportion of the cases has been observed by us ; as to the remainder, we obtained the necessary data from the cards of the cases put at our disposal by various hospitals of the country. The material comprises all types of carcinoma except that of the skin. Sarcoma is not included for lack of sufficient number of cases. Tables I and II refer to all cases ; they indicate the incidence of hypertension found in cancer patients arranged only according to sex and age of the patient.

TABLE I.—*Carcinoma in Men* (579 *Cases*).

| Age-group. | Number of cases. | Hypertension found (%). | Usual incidence of hypertension (%).* |
|---|---|---|---|
| 40–49 | 100 | 3·0 | 10·3–12·6 |
| 50–59 | 178 | 5·0 | 20·5–22·0 |
| 60–69 | 215 | 14·0 | 26·6–30·0 |
| 70 and more | 86 | 12·8 | 32·6–30·0 |

* In all tables the first figure is that of Master, Marks and Dack (1943), the second of the writer.

TABLE II.—*Carcinoma in Women* (911 *Cases*).

| Age-group. | Number of cases. | Hypertension found (%). | Usual incidence of hypertension (%). |
|---|---|---|---|
| 40–49 | 289 | 7·4 | 13·3–16·7 |
| 50–59 | 307 | 22·1 | 24·0–31·0 |
| 60–69 | 251 | 33·8 | 29·0–42·0 |
| 70 and more | 64 | 50·0 | 34·0–53·0 |

As can be seen, there is a great difference between men and women. In women with carcinoma the rate found for essential hypertension does not deviate to any great extent from the normal figure of hypertension morbidity ; only in the age-group 40 to 49 is the percentage smaller, i.e., 7·4 compared to the normal figure of 13·3 to 16·7. In men, on the other hand, the deviation from the normal rate is much greater and applies to all age-groups, especially to the two age-groups 40 to 49, 50 to 59 ; in the group 40 to 49 essential hypertension is met with in 3·0 per cent instead of the normal morbidity of 10·3 to 12·6 per cent, and in the age-group 50 to 59 it is noted in 5·0 per cent instead of the expected figure of 20·5 to 22·0 per cent.

Do the various types of carcinoma behave in a different way ? Tables III, IV and V reproduce the findings in women suffering from carcinoma of the breast, the genitals (ovaries, uterus and vagina), and of carcinoma of the digestive tract (oesophagus, stomach, large intestine and rectum).

TABLE III.—*Carcinoma of Breast in Women* (340 *Cases*).

| Age-group. | Number of cases. | Hypertension found (%). | Usual incidence of hypertension (%). |
|---|---|---|---|
| 40–49 | 161 | 8·7 | 13·3–16·7 |
| 50–59 | 105 | 22·8 | 24·0–31·0 |
| 60–69 | 62 | 30·6 | 29·0–42·0 |
| 70 and more | 12 | 50·0 | 34·0–53·0 |

TABLE IV.—*Carcinoma of Genitals in Women* (222 *Cases*).

| Age-group. | Number of cases. | Hypertension found (%). | Usual incidence of hypertension (%). |
|---|---|---|---|
| 40–49 | 66 | 7·6 | 13·3–16·7 |
| 50–59 | 87 | 32·2 | 24·0–31·0 |
| 60–69 | 53 | 41·5 | 29·0–42·0 |
| 70 and more | 16 | 50·0 | 34·0–53·0 |

TABLE V.—*Carcinoma of Intestinal Tract in Women* (222 *Cases*).

| Age-group. | Number of cases. | Hypertension found (%). | Usual incidence of hypertension (%). |
|---|---|---|---|
| 40–49 | 37 | 2·7 | 13·3–16·7 |
| 50–59 | 70 | 17·1 | 24·0–31·0 |
| 60–69 | 88 | 30·0 | 29·0–42·0 |
| 70 and more | 27 | 48·0 | 34·0–53·0 |

TABLE VI.—*Carcinoma of Intestinal Tract in Men* (379 *Cases*).

| Age-group. | Number of cases. | Hypertension found (%). | Usual incidence of hypertension (%). |
|---|---|---|---|
| 40–49 | 64 | 1·6 | 10·3–12·6 |
| 50–59 | 111 | 3·6 | 20·5–22·0 |
| 60–69 | 145 | 14·5 | 26·6–30·0 |
| 70 or more | 59 | 10·1 | 32·6–30·0 |

In carcinoma of the breast, as well as that of the genitals, the incidence of hypertension almost equals the normal figure ; a deviation is to be observed only in the first decade. So far the picture is the same as that reproduced in the general statistics (Table II). In carcinoma of the digestive tract, however, the deviation from the normal figure of hypertension-morbidity seems to be more marked, and not only to be found with regard to the age-group 40 to 49, but to the

50 to 59 group also, though the deviation in the latter group is only of a minor degree (Table V). The number of other types of carcinoma, such as carcinoma of the gall-bladder, pancreas, liver, kidneys and respiratory tract, was not great enough to be used for separate statistical elaboration. For the same reason, in men, only the cases of carcinoma of the digestive tract were statistically elaborated.

Essential hypertension proved to be a rather infrequent occurrence in all age-groups, but especially between the ages of 40 to 59. It is more or less the same picture as that reproduced in the general statistics (Table I).

Though the number of cases of carcinoma of the respiratory tract examined by us was relatively small, we tended to find the same relationship to hypertension as in Table I. We examined 37 cases in the age-group 40 to 59, of whom only one showed hypertension.

Two factors may be held responsible for the rare occurrence of a combination of carcinoma and essential hypertension : (1) carcinoma may have a depressing influence on a previously existing hypertension ; (2) a person suffering from essential hypertension may have smaller tendency to develop carcinoma.

The possibility of a depressing action of carcinoma cannot be denied. In this connection we may quote the investigations of Rosenfeld (1929), Feldweg (1929) and Fortunati (1936), who were among the few who carried out systematic blood-pressure investigations in cases of cancer ; in the course of the illness a fall in blood pressure could be detected, though the fall, if present, was in most cases only moderate. The examinations took into consideration the systolic pressure only. The number of cases examined by Fortunati (1936) was very small ; yet it occurred to him that high blood pressure is more often met with in cases of cancer of breast and female genitals than in cases of cancer of the digestive tract ; he failed, however, to take into consideration the normal incidence of hypertension ; therefore, he could not even try to answer the decisive question as to whether the frequent occurrence of hypertension in carcinoma of the breast and female genitals or its rare occurrence in carcinoma of the digestive tract are to be considered as extraordinary. A depressant action on blood pressure by carcinoma, as formulated by the authors mentioned above, might be attributed to non-specific factors such as inanition, anaemia, raised temperature and even imposed rest of long duration. It seems unlikely, however, that these factors could be held responsible in any great degree for the rather rare occurrence of carcinoma combined with hypertension in men ; after all, in women suffering from carcinoma of the breast and genitals essential hypertension appears in almost its usual frequency, although the non-specific factors mentioned above certainly also constitute part of their disease. Moreover, in order to eliminate these factors as much as possible, we excluded all carcinoma cases with normal or even subnormal blood pressure, if the carcinoma was in an advanced stage or complicated by raised temperature. Especially in men, only those cases were registered whose general condition was good enough to justify at least the trial of a radical surgical treatment ; this held true particularly with regard to the cases of carcinoma of the digestive tract. Patients with normal blood pressure but in a less satisfactory general condition were also registered if either the blood pressure existing before the manifestation of cancer was known to be normal or other symptoms such as size of the heart, electrocardiogram and eye fundus were not of such a nature as to arouse the suspicion of a previous hypertension. It must be stressed, however, that both the X-ray examination of the heart and the electrocardiogram have a restricted value only, since, as it is known, signs of left ventricular hypertrophy may sometimes only be seen after hypertension has been existing for many years. On the other hand, left ventricular hypertrophy does not always allow us to diagnose "essential hypertension," since common arteriosclerosis without hypertension, too, may cause such changes, especially if the people concerned are over 50 or even 60 years of age (Bell, 1946 ; Fishberg, 1939). Carcinoma cases with hypertension were, of course, always registered, no matter whether their general condition was good or not.

So far only non-specific symptoms accompanying cancer disease such as cachexia, etc., have been taken into account as factors which might exert a negative action on blood pressure. Another question before us is whether cancer itself may act in a depressive sense upon blood pressure by means of specific substances possibly present in its tissue or of catabolics appearing in the cancer patient. If this were the case, it would not be easily understood why hypertension in women with carcinoma of breast or genitals is met with in nearly normal frequency ; one could, perhaps, argue that antipressor substances do not necessarily appear in every type of cancer. It is clear, however, that the question as to whether hypertension may be influenced by cancer can unequivocably be answered only by examinations aimed at determining the blood pressure before cancer manifested itself. In our examinations this was possible only in a small proportion of the cases. If only one blood-pressure examination could be secured from the past it was disregarded if it was carried out less than one year or more than three years before the manifestation of the cancer disease. Table VII indicates the percentage of men who suffered from essential hypertension before cancer developed. In view of the small number of cases examined, no differentiation into the various types of cancer seemed advisable; the majority of the cases belonged, however, to the group of cancer of the digestive tract. Moreover, the age-groups were clumped together in two age-groups only.

TABLE VII.

| Age-group. | Number of cases. | From hypertension suffered in the past (%). | Usual incidence of hypertension (%). |
|---|---|---|---|
| 40–59 | 72 | 4·2 | 15·4–17·3 |
| 60 and more | 106 | 21·6 | 29·6–30·0 |

As can be seen, men falling ill with carcinoma between the ages 40 to 59 seldom displayed the symptoms of essential hypertension before the onset of the disease ; as to the age-groups of over 60 years the result was less conspicuous. This material is certainly not great enough for drawing far-reaching conclusions ; it does not allow us to decide whether the rare occurrence of hypertension in certain types of carcinoma and in certain age-groups is attributable alone to a lesser inclination to cancer of people with hypertension, or to a negative mutual correlation which to some extent may also render possible an influence of cancer on hypertension. It is, in my opinion, this research—the investigation of blood-pressure conditions existing before manifestation of cancer disease—which will have to be put on a very broad basis in future.

Essential hypertension and common arteriosclerosis are recognised as quite different processes. In the first years of essential hypertension any anatomical change typical of arteriosclerosis may be absent and, on the other hand, very severe arteriosclerosis may be found in people with normal or even subnormal blood pressure. Essential hypertension, however, is known as one of the diseases which, like diabetes mellitus, predisposes to the development of arteriosclerosis. If our assumption is correct, that people with normal blood pressure have greater inclination to certain types of cancer, severe arteriosclerosis may be met with less often in cancer patients than in other people. In this connection it may be of interest to learn that Casper as long ago as 1932 noticed that in people with gastric carcinoma the aorta is very often of a character which one would expect to find only in young people ; and while drawing up this text I came across the lately published paper of Wanscher, Clemmeson and Nielsen (1951), who on the strength of systematic anatomical examinations also came to the conclusion that arteriosclerotic lesions are less pronounced in patients suffering from cancer than among non-cancerous persons. As it is known, a much closer correlation exists between hypertension and arteriolosclerosis than between hypertention and arteriosclerosis. Therefore, anatomical examinations purposed to determine the correlation between cancer and arteriolosclerosis might have an even more definite result than those concerning the connection between cancer and arteriosclerosis. According to our results we should expect that the incidence of arteriolosclerosis in cancer patients, and particularly in male patients aged 40 to 59, would be much lower than in other people of the same age.

### DISCUSSION.

Simple as the diagnosis of essential hypertension is in evident cases, it may be equally difficult in borderline cases. It might be considered as arbitrary to regard a blood pressure of more than 160/90 mm. Hg as the minimum value for essential hypertension ; but any other limit is hardly less arbitrary. There are authors who consider a pressure as low as 150/90 mm. Hg as minimum value, in which case the number of cancer patients with hypertension would certainly be greater ; but the figure for normal hypertension-morbidity would accordingly increase, and it is hardly to be assumed that a change in the mutual relationship would result.

We feel that our investigations justify the assumption that people suffering from essential hypertension have a lesser inclination to carcinoma ; but we have certainly not put forward any reason for this phenomenon, and any attempt to search for a satisfactory explanation must encounter great difficulties, among which the following may be mentioned : (1) the aetiology of essential hypertension is by no means clear ; it is even not certain whether it has a unitary background ; (2) the fact that in people with hypertension certain types of cancer or cancer of certain age-groups are only seldom met with does not prove that it is the hypertension itself, i.e., its underlying pathological process, which either checks development of cancer or—as can be also inferred from our examinations—delays its manifestation for years or even decades. Possibly an unknown constitutional factor may play a part which is favourable for the development of hypertension but not for cancer or *vice versa*. The possibility, however, that it is the pathological process underlying hypertension which directly checks development of cancer cannot be excluded. Does, perhaps, a competitive action of pressor- and carcinogenic substances come into question ? In view of our present entirely insufficient knowledge of existence and nature of these substances (steroids ?) we may be permitted to put this question, but we can certainly not expect an answer as yet. Nor is there any answer to the question why the negative correlation between essential hypertension and cancer is more marked in men than in women, especially those suffering from cancer of the breast or genitals. At any rate, at the present stage of our investigations it does not seem practical to search for satisfactory explanations ; after all, the purpose of this report is only to draw attention to certain facts which are in need of further expansion and completion.

### SUMMARY.

(1) The incidence of essential hypertension in people suffering from carcinoma is lower than in other people of the same age-group.

(2) The deviation from the usual hypertension rate is more marked in men than in women, and apparently more so in women suffering from carcinoma of the digestive tract than in those with carcinoma of the breast or genitals.

(3) The possible nature of the negative correlation between the two diseases is discussed.

### REFERENCES.

BELL, E. T.—(1946) ' Renal Diseases.' London (Henry Kimpton).

*Idem* AND CLAWSON, B. J.—(1928) *Arch. Path.*, **5**, 939.

CASPER, J.—(1932) *Z. Krebsforsch.*, **36**, 354.

FAHR, G.—(1928) *Amer. J. med. Sci.*, **175**, 453.

FELDWEG, P.—(1929) *Münch. med. Wschr.* **48**, 2005.

FISHBERG, A. M.—(1939) ' Hypertension and Nephritis.' Philadelphia (Lea & Febiger), 228.

FORTUNATI, I.—(1936) *Boll. Lega ital. Cancro*, **10**, 210.

MASTER, A. M., MARKS, H. H., AND DACK, S.—(1943) *J. Amer. med. Ass.*, **121**, 1251.

ROSENFELD, A.—(1929) *Med. Klinik*, 1,871.

VOLHARD, F.—(1931) ' Nieren und ableitende Harnwege, Handb. der Inneren Medizin.' (Julius Springer), 1627.

WANSCHER, O., CLEMMESEN, J., AND NIELSEN, A.—(1951) *Brit. J. Cancer*, **5**, 172.